# Status of the health information system in Ireland and its fitness to support health system performance assessment: a multimethod assessment based on stakeholder involvement

**DOI:** 10.1186/s12961-022-00931-1

**Published:** 2022-11-16

**Authors:** Damir Ivanković, Tessa Jansen, Erica Barbazza, Óscar Brito Fernandes, Niek Klazinga, Dionne Kringos

**Affiliations:** 1 Department of Public and Occupational Health, Amsterdam UMC Location University of Amsterdam, Meibergdreef 9, Amsterdam, The Netherlands; 2 Quality of Care, Public Health Research Institute, Amsterdam, The Netherlands; 3 Netherlands Institute for Health Services Research (Nivel), Utrecht, The Netherlands; 4 Department of Health Economics, Corvinus University of Budapest, Budapest, Hungary

**Keywords:** Health system performance assessment, Health information system, Assessment, Stakeholder involvement, Ireland

## Abstract

**Background:**

Between 2019 and 2021, the first Irish health system performance assessment (HSPA) framework was developed. As routinely collected health data are necessary to continuously populate indicators of an HSPA framework, a purpose-driven assessment of the health information system (HIS) in Ireland and its fitness to support the implementation of an HSPA framework was conducted. This study reports on the status of the Irish HIS through a multimethod assessment based on continuous broad stakeholder involvement.

**Methods:**

Between May and November 2020, over 50 informants were engaged in individual and group interviews and stakeholder consultation workshops as part of the HIS assessment process. Descriptive themes and high-level data availability heatmaps were derived from interview and workshop data using thematic analysis. Indicator “passports” for the HSPA framework were populated during stakeholder consultation workshops and analysed using univariate descriptive statistics.

**Results:**

The HIS in Ireland was able to provide administrative, survey and registry-based data for public sector acute care services, focusing on structure, process and output metrics. Significant data availability gaps, most notably from primary care, private hospitals and community care, were reported, with little availability of electronic health record and people-reported data. Data on outcome metrics were mostly missing, as were linkage possibilities across datasets for care pathway monitoring. The COVID-19 pandemic highlighted the national HIS’s shortcomings but also the capacity for rapid development and improvement.

**Conclusions:**

A tailor-made assessment of the HIS in Ireland, involving a broad set of relevant stakeholders, revealed strengths, weaknesses and areas for improvement in the Irish health data landscape. It also contributed to the development of a national HSPA framework and momentum to further strengthen data infrastructure and governance, while working towards a more data-driven and person-centred healthcare system. This work demonstrates the utility of an inclusive HIS assessment process and is applicable beyond Ireland, where this case study was conducted.

**Supplementary Information:**

The online version contains supplementary material available at 10.1186/s12961-022-00931-1.

## Background

Data are crucial in understanding how a health system and its services are performing [1]. When collected on an ongoing basis and fed into the delivery of health and social care, these data are referred to as the health information system (HIS) [2] and are considered one of the six main building blocks of any health system [3]. Only when turned into information and ultimately performance intelligence [4] can HIS data can help manage and improve the health system’s central role of providing accessible, quality and safe care and maintaining and enhancing individual and population health. Over the last two decades, health system performance assessment (HSPA) has emerged as a central method and tool for reporting and using performance intelligence needed to monitor, manage and improve national health systems, as well as to achieve alignment with strategic policy goals and aims [5, 6]. As mentioned, the HSPA framework’s indicators are populated from routinely collected HIS data. Ideally, national data infrastructure and governance mechanisms ensure sufficient data availability, timeliness, accuracy, completeness, usability and relevance [7], including data linkage possibilities across datasets, covering various health services, using different data sources and spanning data types.

**Fig. 1:**
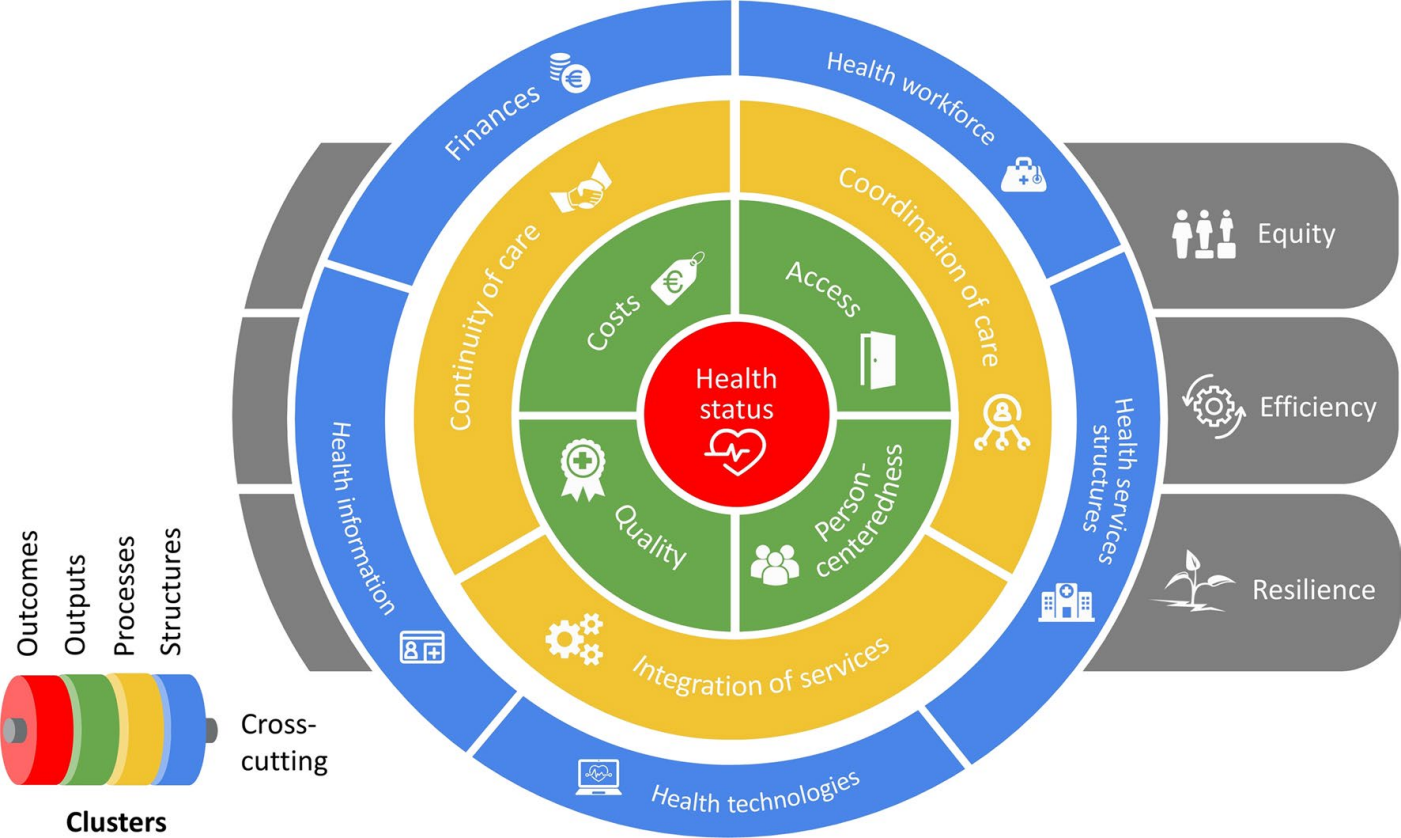
Graphical display of indicator clusters and domains in the proposed Irish HSPA framework (2021)

In 2017, a major 10-year health reform called Sláintecare was launched in Ireland [8]. To measure progress in achieving reform objectives and their alignment with the broader policy cycle, the Irish Department of Health (DoH) and the Health Service Executive (HSE) initiated and supported the development of an *actionable* [9] national HSPA framework for assessing health system governance and performance. The development of the framework was supported through the European Union’s (EU) structural support reform programme [10], and led by an external team of researchers through the “Performance accountability for the Irish health system” project. At the end of the project, an HSPA framework for Ireland was proposed [11], consisting of five clusters, 16 domains and 36 subdomains, 49 features and 266 indicators (Fig. 1), and was officially launched by the Irish Minister of Health.

In past years, several health information infrastructure and governance assessments have been conducted in Ireland, some of which are also publicly available [12–14]. These were generally stand-alone HIS assessments, not directly linked to broader policy or reform implementation efforts and usually involving a limited number of national stakeholders in the process. The findings of these assessments often emphasized Ireland’s relative “lag” in HIS governance and infrastructure development, especially compared to other western European countries. Suboptimal strategic coherence was described to have led to implementation challenges, accentuated by a fragmented health data landscape. The more recent among these assessments reported on recent or planned developments in national health information strategy and governance [12].

Having recognized the crucial role of routinely collected HIS data in populating an HSPA framework, a tailored assessment of the status of the HIS in Ireland was deemed necessary. To ensure an integrative approach, capacity-building and ownership in a complex interorganizational landscape such as a national HIS, the close involvement of stakeholders in all phases of assessment, development and implementation was pivotal [15, 16].

With an aim to assess the status quo of the national HIS and its fitness to support the development and implementation of the first HSPA framework in Ireland in 2020, this paper presents the results of a systematic multimethod HIS assessment, based on broad and continuous stakeholder involvement and participation.

## Methods

An iterative, multimethod assessment was conducted in 2020 to describe the status of the HIS in Ireland and assess its fitness to support the development and implementation of the national HSPA framework. The assessment used (i) key informant interviews (*n* = 16 interviews) and (ii) stakeholder consultation workshops (*n* = 6 workshops). First, we descriptively summarized the Irish HIS’s main characteristics and recent developments through stakeholder narratives and data availability mapping across services. Secondly, we specifically investigated the fitness of the HIS to support the implementation of a national HSPA framework in Ireland, by focusing on data availability for clusters and indicators in the proposed HSPA framework and by exploring data linkage possibilities.

The research team included healthcare performance intelligence researchers and practitioners with previous experience working with health data and policy, designing HSPA frameworks and conducting HIS assessments in the European and global context. The research team worked closely with the Irish health authorities, namely the DoH and the HSE, while maintaining full scientific autonomy.

The status of the HIS in Ireland was assessed using interview data and focused on describing its main characteristics and mapping data availability across healthcare services. The fitness of the HIS to support the implementation of a national HSPA framework was assessed by mapping data availability across the five main clusters and 266 indicators of the proposed HSPA framework, including identification of relevant data sources and data custodians. The assessment also took stock of additional HIS-related topics identified as relevant by the stakeholders involved in the HSPA development process. The data availability mapping framework categories were based on the HSE’s classification of health services in Ireland [17], indicator clusters for the proposed HSPA framework (Fig. 1), Sláintecare reform priorities [8] and WHO’s Health Metrics Network HIS assessment tool [18]. The indicator “passport” methodology was developed internally, based on the research team’s experience with developing performance intelligence for primary care [19].

### Key informant interviews

Semi-structured, individual and group key informant interviews were organized and aimed to elicit information from a range of relevant national stakeholders with regard to the HIS and performance measurement in the Irish health system. The research team and the Irish health authorities agreed on conceptualizing stakeholders (informants for this assessment) as people and institutions with potential to influence the outcome of the abovementioned HSPA project. Based on this common understanding of stakeholders, an initial list of potential informants was proposed by the DoH (*n* = 32) and was expanded during the interview process, based on interviewee suggestions, applying a snowballing approach [20]. Broad stakeholder involvement within the Irish health system was achieved, with representation from the HSE, DoH, regulatory and health professional bodies, research institutions, other governmental institutions and patient organizations, as well as other custodians of health data. Between 29 May and 27 October 2020, the study team (DI, TJ, EB, OBF) conducted 16 remote, 1-hour interview sessions involving 18 key informants (10 female, 8 male), representing key stakeholder organizations (Additional file 1, for a list of informants consulted per stakeholder). The interviews discussed the HIS in Ireland, specifically health data, indicators and data sources, as well as performance measurement, monitoring and management practices. Before each interview, informants were provided with preparatory materials and guiding interview questions (Additional file 2). Having received verbal agreement from the informants, interviews were recorded and transcribed. Thematic analysis was used to analyse interview data inductively and deductively and to populate data availability heatmaps and summarize HIS status quo narratives, which are additionally presented through anonymized verbatim quotations [21–24].

### Stakeholder consultation workshops

Six interactive online stakeholder consultation workshops were convened between 15 October and 26 November 2020, with an aim to further assess the quality of the national HIS and health data needed to populate the proposed HSPA framework. Workshops involved over 40 stakeholders from different organizations across the Irish healthcare system, some of whom had also previously participated in the abovementioned interviews. Each workshop aimed to closely review a working list of potential indicators for the HSPA framework, paying particular attention to data availability as well as to the infrastructure and governance in place. Each workshop lasted 2 hours and was attended by between 11 and 16 participants. Workshops were organized around the five main clusters of the proposed HSPA framework (as shown in Fig. 1), with an additional, sixth workshop organized, following a suggestion by the DoH, which specifically focused on indicators related to Sláintecare reform priority policies. In advance of each workshop, “homework” spreadsheets were shared with participants, which listed the indicators identified through parallel research steps of the project and pertaining to the workshop’s cluster of focus (Additional file 3). Spreadsheets contained an indicator “passport” for each indicator, with assessment categories such as the indicator’s fitness for the HSPA framework’s purposes, data availability and methodological quality. An example of a brief and a spreadsheet containing indicator passports is shown in Additional file 3. Spreadsheet data were analysed using univariate descriptive statistics. To assess the fitness of the HIS in Ireland in supporting the development and implementation of a national HSPA framework, we specifically looked at (i) what components of the framework were assessed as measurable or not, and (ii) which data custodians owned and managed the data.

## Results

### The status of the HIS in Ireland

Based on key informant interviews, the HIS in Ireland was, in 2020, generally assessed as being able to provide data that were, when available, of good quality, accuracy, validity and timeliness, including demographic and geographical disaggregation possibilities. However, significant gaps were identified in the completeness of the data, with data mostly being available for publicly provided acute care services and largely missing for services provided in the private sector, notably independent hospitals, general practice and community health centres. For a similar reason, respondents reported good data coverage for the lower-income groups, but much less for the middle-income groups, due to the latter using both public free-of-charge and paid private provider services. Registry data were reported to be readily available and of good quality. This was explained by population and disease registries having long histories and tradition in Ireland, which often meant dedicated and well-established operational and research teams, high levels of attention to data quality, extensive international collaboration and sustainable funding. In line with Sláintecare reform priorities, the use of HIS data to assess and address regional differences in the uptake of policies around Ireland was found crucial. However, informants concluded that the current HIS was not able to fully support these regionalization efforts due to varying levels of data completeness across regions. Additionally, inadequate focus on case-mix and risk-adjusted indicator calculations, despite data being able to support both, hindered its use for benchmarking between regions. Finally, lack of high-level agreement on key priority indicators in the health system, a national oversight body, and consistent and standardized tracking of performance results over time were identified as hindering factors in working with health data in Ireland.

**Fig. 2:**
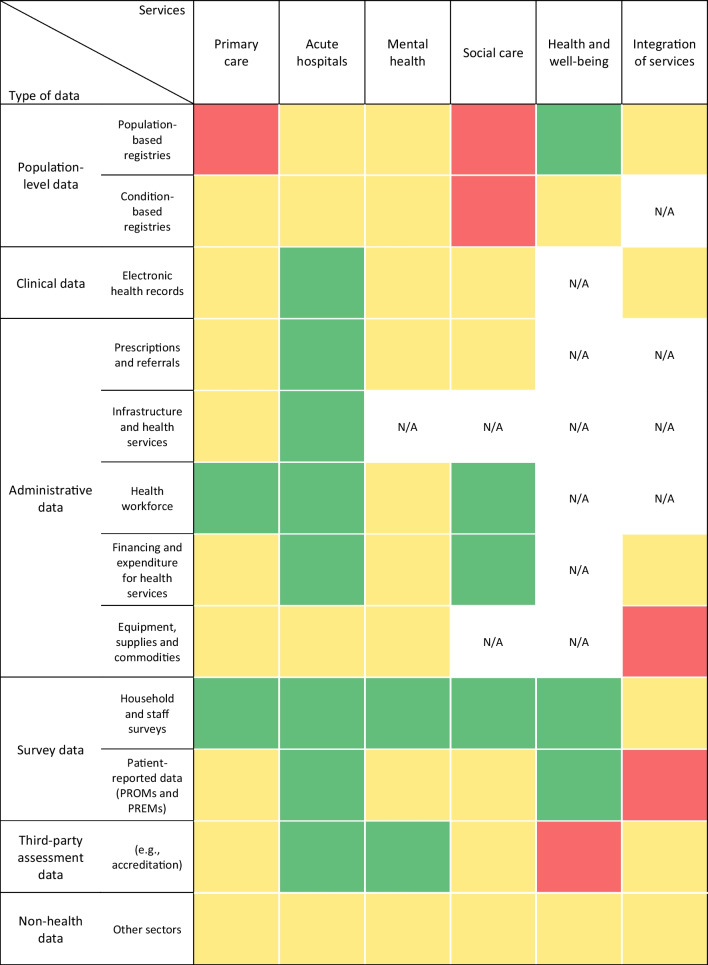
Heatmap of data availability by data sources and main categories of healthcare services. Data availability mapping based on data obtained from the stakeholder interviews. Red = data not available; yellow = data partly available or technical capacity is (probably) available; green = data available; white/N/A = category not applicable or no information on data availability collected during interviews. The acute hospitals category includes only acute public hospitals, as such information is not centrally gathered for private hospitals. The social care category includes long-term care and disability services. Mental health includes inpatient, outpatient and acute mental health services

**Fig. 3:**
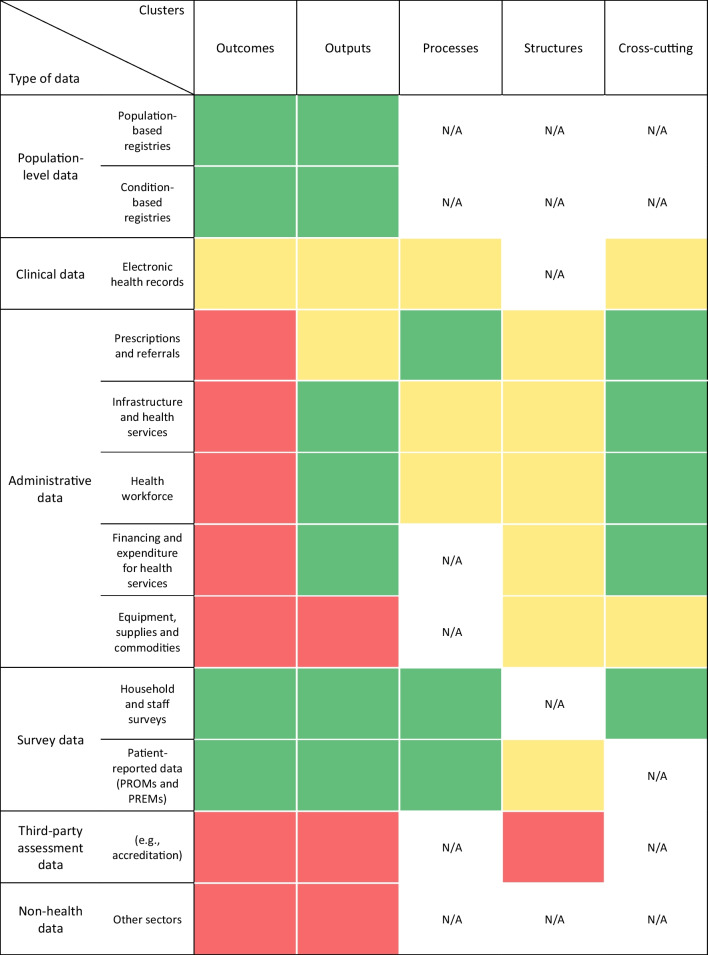
Heatmap of data availability by data sources and the clusters of the proposed HSPA framework. Data availability mapping based on data obtained from the stakeholder interviews. Red = data not available; yellow = data partly available or technical capacity is (probably) available; green = data available; white/N/A = category not applicable or no information on data availability collected during interviews

Analysis of information collected through stakeholder interviews also enabled mapping data availability across the healthcare system’s services (Fig. 2). Data were described as generally readily available for acute hospital care services across different types of data. Survey-sourced data from the annual Healthy Ireland survey were also obtainable across many services. Administrative data, especially those on health workforce and financing health services, were available for most acute, social and primary care services. For many assessed categories, the data were found to be “partly available or the technical capacity is (probably) available”, as depicted by yellow cells in Figs. 2 and 3. This finding reflects a need for improvements to data collection or analytics processes in order to use these data for indicators in the HSPA framework. In contrast to categories where data were found “not available” (red cells), cells labelled yellow indicate that new data collection systems do not need to be set up.

### The fitness of the HIS to support implementation of a national HSPA framework

Based on interview data and using the same categories of data types as in the previous heatmap, the five main clusters of indicators proposed for the Irish HSPA framework were mapped for data availability (Fig. 3). The most notable finding was generally lower availability of data pertaining to the health outcomes and cross-cutting indicator clusters of the HSPA framework. Due to the Irish HIS’s focus on structures, processes and outputs, at the time of the assessment, informants generally assessed its data as inadequate in supporting the framework’s focus on outcomes (and linking outcomes to inputs). The HIS was also assessed as suboptimal for providing data on the adaptability, resilience and up- and down-scaling capacity of the system, including its infrastructure, services and workforce.

When discussing the HIS in Ireland in relation to its ability to populate the proposed HSPA framework, key informants pointed to three additional areas of interest, not originally included in the listed interview topics: (i) data linkage and the ability to analyse and manage care pathways and integration of care, (ii) collection, reporting and the use of people-reported data, and (iii) recent developments, as a direct and indirect consequence of the ongoing (at the time of conducting this work) COVID-19 pandemic.

Pathways of care were not well captured in data, and the data to measure care integration were mostly unavailable. Informants mainly attributed this to suboptimal data linkage efforts which, instead of enabling patients to be followed through the system, mostly inferred care pathways and subsequent correlations. Slow implementation of the individual health identifier (IHI) and historical use of proprietary data standards hindered data linkage capabilities. Informants also attributed the limited data linkage capabilities to the lack of a dedicated national health data coordination body and a coherent strategic approach to health data. Proxies were used, such as bed days by people with primary care sensitive conditions, but it was pointed out by informants that it is impossible to have integrated care without fully integrated data. However, attitudes towards data use and sharing among stakeholders in the system have recently changed, with a newfound appreciation for data use for policy, by policy- and decision-makers. Primary care was exemplified as an area in which an improved flow of data between general practitioners, hospitals and the HSE would lead to better coordination and integration of care. Lack of clinical data flow between most community service providers and the HSE was also emphasized as a shortcoming. Other causes and examples of data linkage issues were presented, such as unaligned data standards and technical solutions among private hospitals with different systems for patient data collection and reporting and subsequently non-interoperable information systems.

According to informants, there seemed to be increasing interest and recognized need for the collection of people-reported data in the Irish health system. This included both patient-reported outcome and experience measures (PROMs and PREMs) and staff- and carer-reported data. However, apart from the annual Healthy Ireland survey for some services and a few smaller pilot projects, these kinds of data were not structurally collected, reported or used. Collecting people-reported data was seen as challenging and, hence, often done in small, research-focused ways rather than consistently by healthcare organizations as a way of learning from patients, staff and carers. Focusing these qualitative metrics on issues that matter to people rather than on questions such as satisfaction with the cleanliness of the hospital was another theme, often repeated by informants. So was the lack of patient experience measures in primary care. Person-centeredness was recognized as key to patients and citizens, with suggestions by informants for Ireland to join ongoing international initiatives, developing this area further.

With this assessment being conducted during the COVID-19 pandemic, issues of health data management during a public health emergency were also mentioned by informants. They agreed that it was difficult to say whether changes to the data landscape, catalysed by the pandemic situation, would persist, but that it was necessary to consider the potential of these developments. For instance, a temporary, emergency version of the IHI was rolled out to follow patients with COVID-19 through the system and for vaccination purposes [25, 26]. Also, a novel emergency data hub for researchers to access data about COVID-19 was established, where data could be linked and accessed through the Central Statistics Office’s infrastructure [27]. According to informants, the views on how data are collected, accessed, reported and used have changed due to the pandemic. The crisis highlighted both the system’s shortcomings and its strengths. These improvements made data for acute care of COVID-19 readily available on a very granular level across organizations. However, the quality of non-COVID-19 care data had not improved and, some informants felt, might have even worsened. Questions of data quality, with such rapid data infrastructure developments, were raised, and informants noted that, due to the urgency, less attention was put to the minimum requirements of datasets. In general, informants were hopeful that the positive developments would be sustained after the pandemic (Table 1).

**Table 1:** Selected illustrative quotes by interview informants

Focus on	Illustrative verbatim quotations
Status quo of the Irish HIS	• *One of the things that became obvious to us right from the start of this is that we have quite good data systems on the acute side. But when it comes to the community side of service provision, or privately provided service, they are much less well developed and much more scattered. And this might be something that you have seen in other areas as well*. (1) • *And so, I do think that there is a frustration with respect to health information at the ground level associated with a lack of centralized thinking about health data and access to it. So, I think we need a singular entity, as a single office, that is given the remit to oversee health information management, and that would include standardization of data sets, KPIs [key performance indicators] and initiatives. That doesn’t mean they have to manage it, but that they would oversee it from a quality point of view or standards point of view*. (5) • *The nursing homes data is good. That’s because it’s a policy that’s gone back 10 years now. It’s particularly good disaggregation, such as age, gender, location, average length of stay… that’s brilliant. But then there are other areas of health data that don’t have that coverage*. (3) • *The key policy driver here, also around health data, currently is the Sláintecare programme. And that’s pretty much the only show in town*. (5)
Data linkages and care pathway monitoring	• *When a patient goes through the system, it is not something that is collected, or should I say connected. Pathways of care are not captured in data*. (2) • *Access to data is probably the biggest problem we have in the Irish health system right now, who actually owns it. Also, there is no connection or sharing or flowing of information, whether that be from the acute to the community sector, or even within the community sector*. (3) • *It’s difficult to measure outcome measures when you don’t have a data infrastructure that allows you to do that, especially not to link episodes of care*. (2)
People-reported data	• *There’s certainly some, like patient experience survey. We would see that as a good model of data collection and accessibility for both the policy-maker, the service provider and the public. And we’d like to see more expansion in a similar kind of way, I suppose. Yeah, it has, I suppose it has its own challenges, and that it was a model that had to be developed. But it does lend itself to good governance, to transparency to everybody knowing what they can use the data for. And I think that’s, I think that's really, really important*. (4) • *So, there’s the national inpatient experience survey, which is now well established. And that has been through a couple of annual cycles. It’s usually repeated every other year. So, this year, it got paused, because obviously, it wasn't appropriate to run it in the middle of pandemic, but hopefully it will continue next year*. (5)
Role of the COVID-19 pandemic	• *COVID-19 has actually forced an awful lot of people to re-evaluate how they interact with the health system, because nobody wants to go to the hospital, and this has huge data implications*. (5) • *So, you know, health professionals in the system are becoming more competitive due to COVID-19 and they’re wanting to know more. Also, how they compare, as opposed to just looking at their organization*. (2)

Quotes are anonymized. Numbers next to quotes denote informants’ organization type, as follows: (1) = national health authorities and other governmental institutions; (2) = regulatory and health professional bodies; (3) = research/academic institutions; (4) = patient organizations; (5) = other custodians of health data

**Fig. 4:**
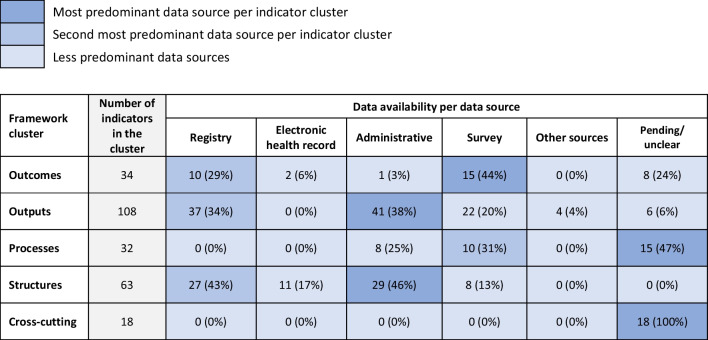
Data availability by types of data sources and indicator clusters of the proposed HSPA framework. Availability of data based on indicator “passports” populated before and during stakeholder consultation workshops. Sums of all data sources across clusters are not equal to the total number of indicators in each cluster. Some indicators might be populated with data from multiple data sources. Pending/unclear = data availability and data source for the proposed indicator requires further clarification or is in the process of being set up

Following pre-workshop preparatory work by stakeholders on indicator passports and discussions during all six workshops, a total of 266 HSPA indicators were assessed as potentially useful for the first Irish HSPA framework. Data availability and potential data sources were assessed for each. Differing levels of data availability across the five indicator clusters were found, as shown in Fig. 4. Data for the proposed framework were mostly available for indicators in the structures and outputs cluster and sourced from administrative, registry and survey data. Data sourced from electronic health records (EHRs) were able to populate only 0.5% of all proposed indicators (13/266).

Data for the 266 potential indicators of the proposed HSPA framework were available from various sources and managed by different data custodians within the Irish health system. Based on the results of stakeholder consultation workshops, two organizations were responsible for most of the indicator data. One quarter of all proposed indicators (72/266, 27%) were available from datasets held by the HSE, namely data collected and managed by the National Quality Improvement Team as well as the Hospital In-Patient Enquiry, National Patient Experience Study and the Healthy Ireland survey datasets. Another 18% of indicators were available from data managed by the Central Statistics Office, and its annual Survey on Income and Living Conditions dataset. Other relevant data custodians and datasets included the Health Information and Quality Authority (14/266, 5%), Irish Cancer Society (12/266, 5%), Higher Education Authority (9/266, 3%), National Vaccine Information System (6/266, 2%) and the Health Research Board (5/266, 2%). Data to populate nearly a third of proposed indicators required further clarification of custodianship and sources (79/266, 30%).

This assessment also identified several specific data source type-related challenges. The coverage and timeliness of data collected primarily for administrative purposes was generally good, especially for acute care services, but its usefulness for monitoring population-level health and individual patient-level outcomes, which are important for an HSPA framework, remained limited. The use of population and patient survey data in Ireland has been increasing in prevalence and importance. At the time of the assessment, it covered many of the services, as well as clusters and domains conceptualized in the proposed HSPA framework. Ad hoc PROMs and PREMs collection efforts generally could not fully support the envisioned framework and its focus on the use of indicators based on patient-reported data. Despite recent localized rollouts of new standardized EHRs among acute care services in Ireland, this modality of capturing health data was still very limited in its linkage and reuse capabilities. Legal requirements to adopt EHRs and adhere to standards were listed as some of the possible reasons. The increasingly important role of software solution vendors was also mentioned in both interview and workshop stages of this assessment.

## Discussion

With this study, we assessed the status of the HIS in Ireland in 2020 and its fitness to support the development and implementation of an HSPA framework. Our findings identified HIS’s strengths, weaknesses and areas for improvement, while immediately providing input for the development of the first Irish HSPA framework.

This work was conducted using a novel HSPA-focused HIS assessment methodology, which emphasized continuous and broad stakeholder involvement. The assessment’s focus on the aspects of HIS used to populate an HSPA framework allowed for a more streamlined process, directly contributing to the HSPA framework development and implementation as well as signalling future HIS development areas for this purpose. Continuous involvement of a broad range of stakeholders and a mixed qualitative and quantitative methodology allowed for the collection of relevant and highly contextually loaded information. Similar to other research activities in the broader HSPA project, which revealed substantial motivation of the Irish health system stewards in strengthening citizens’ voice through shared priority-setting [28], the HIS assessment process also contributed to the general awareness, support and sense of ownership of the broader HSPA project among stakeholders.

High-level HIS assessment findings revealed a national HIS capable of providing relevant data of perceived high quality for acute care services provided in the public sector, mostly sourced from administrative, survey and registry data and focusing on structure, process and output measures. Shortcomings and areas for improvement predominantly related to significant data availability gaps, most notably from primary care, private hospitals and community care. The availability of EHR and people-reported data was suboptimal, as was the availability of data on outcome measures. At the time of this assessment, data linkages across data custodians, data sources and types of data were limited, inhibiting care pathway mapping and better integration of care. Our findings, for the most part, matched the findings of previous HIS assessments in Ireland. However, recent Sláintecare reform processes and the COVID-19 pandemic emphasized some of the identified shortcomings but also revealed the system’s potential to rapidly innovate and improve. Localized rollouts of a new, standardized EHR for publicly provided acute care services in Ireland [29], supported by recent strategic and technical [30, 31] initiatives on a national level, have been reported. The EU’s recent European Health Data Space (EHDS) initiative and international comparative research work have highlighted the relevance of legal requirements for adopting EHRs and adhering to data standards, supported by both national and pan-European strategies and coordination, in helping facilitate better secondary use of health data [32–34]. Stakeholder experiences also signalled that the increasingly important role of software solution vendors should be carefully considered and leveraged as well, as it influences data quality, performance of services and user experience [35–37]. Research has shown that the standards on interoperability of various EHR systems should be implemented broadly to enhance further digitalization of healthcare and facilitate broader data exchange [25, 32] but also that the single most important factor for successful implementation of a national EHR system is stakeholder involvement and buy-in [16, 38, 39].

Data privacy and security issues were rarely discussed during this assessment work on the Irish HIS landscape and its role in the HSPA process. Most data needed for an HSPA framework are, in fact, sourced from existing primary data sources, which provides unique opportunities for secondary data reuse but also presents a set of challenges related to data security and privacy. This finding is especially important in the light of the May 2021 ransomware cyberattack on the Irish health system, which both caused prolonged interruption of care provision in the Irish health system and affected the majority of its data services [40, 41].

## Strengths and limitations

The broad, representative and continuous stakeholder involvement approach significantly added to capturing multiple perspectives and increasing the sense of ownership, increasing the likelihood of successful implementation [42]. The process was supported and sponsored by the national health authorities and linked to the ongoing health system reform. The study also carries with it a number of limitations. Close involvement of the Irish health authorities in the process might have introduced bias to the selection of participating informants and their perceived freedom to express personal and professional opinions. By employing a snowballing approach to further identify and recruit interviewees, we attempted to minimize bias. The research period coincided with the COVID-19 pandemic, which meant that the stakeholders’ focus on the topic and their availability was somewhat limited, and potentially skewed participation toward informants more interested and readily available. Conducting this research through teleconferencing allowed for flexible scheduling of shorter online meetings, thus lowering the threshold to participate. Also, participation through both the interviews and subsequent workshops allowed for multiple opportunities to engage. Finally, the HSPA focus of this HIS assessment might hinder its applicability for other uses. As this is a primarily exploratory study, the data sources have not been directly accessed or assessed for quality. This work was focused on maximizing the applicability of findings for this specific purpose but also warrants further quantitative methods to assess the completeness and other components of data quality.

## Conclusions

This tailor-made assessment of the HIS in Ireland, conducted as part of the development of a national HSPA framework, involved a broad set of stakeholders, described the status quo and revealed strengths, weaknesses and areas for improvement in the Irish health data landscape. Such efforts, especially in a dynamic environment, including the ongoing Sláintecare reform, EU’s EHDS initiative and the COVID-19 pandemic, present a window of opportunity for further advancements needed to effectively work towards a data-driven and people-centred healthcare system in Ireland. Finally, this work demonstrates the utility of conducting an inclusive HIS assessment process and is applicable beyond Ireland, where this case study was conducted.

## Supplementary Information

**Additional file 1.** Key informant interviews—Details on interviewed informants. Detailed and anonymized information on informants interviewed for this study

**Additional file 2.** Key informant interviews—Brief. Preparatory brief document sent in advance to interview participants.

**Additional file 3.** Stakeholder consultation workshops—Stakeholder brief and the preparatory document (excerpt). Preparatory brief document sent in advance to participants of stakeholder workshops and an excerpt of a working table used in advance and during these workshops

## Data Availability

The datasets generated and/or analysed during the current study are available from the corresponding author on reasonable request.

## Abbreviations

HIS: Health information system
HSPA: Health system performance assessment
EU: European Union
DoH: Department of Health
HSE: Health Service Executive
EHR: Electronic health record
IHI: Individual health identifier
PROMs and PREMs: Patient-reported outcome and experience measures
EHDS: European Health Data Space
