# Supplementary material for: Status of the health information system in Ireland and its fitness to support health system performance assessment: a multimethod assessment based on stakeholder involvement
Source: Health Res Policy Syst. 2022 Nov 16;21:1. doi: 10.1186/s12961-022-00931-1 (PMC9670073; doi:10.1186/s12961-022-00931-1)
Supplement: Supplementary file 1 — Additional file 1. Key informant interviews—Details on interviewed informants. Detailed and anonymized information on informants interviewed for this study [file 12961_2022_931_MOESM1_ESM.docx]

List of key informants interviewed

| ID | **Organisation** | **Male** | **Female** | **Date** |
| --- | --- | --- | --- | --- |
| 1 | Department of Health |  | 1 | 29.5.2020 |
| 2 | Department of Health | 1 |  | 29.5.2020 |
| 3 | Health Service Executive |  | 1 | 3.6.2020 |
| 4 | Department of Health | 1 |  | 15.6.2020 |
| 5 | Economic and Social Research Institute | 1 |  | 10.7.2020 |
| 6 | Health Service Executive | 1 |  | 22.7.2020 |
| 7 | Royal College of Surgeons | 1 |  | 22.7.2020 |
| 8 | Mental Health Commission |  | 1 | 22.7.2020 |
| 9 | Data Protection Commission | 1 |  | 21.8.2020 |
| 10 | University College Cork | 1 |  | 8.9.2020 |
| 11 | Irish Platform for Patient Organisations, Science & Industry | 1 |  | 10.9.2020 |
| 12 | National Treasury Management Agency |  | 1 | 14.9.2020 |
| 13 | Department of Public Expenditure and Reform |  | 1 | 16.9.2020 |
| 14; 15 | Nursing and Midwifery Board of Ireland |  | 2 | 21.9.2020 |
| 16 | Department of Health, National Patient Safety Office |  | 1 | 13.10.2020 |
| 17; 18 | Department of Health, National Patient Safety Office |  | 2 | 27.10.2020 |
