# Supplementary material for: Status of the health information system in Ireland and its fitness to support health system performance assessment: a multimethod assessment based on stakeholder involvement
Source: Health Res Policy Syst. 2022 Nov 16;21:1. doi: 10.1186/s12961-022-00931-1 (PMC9670073; doi:10.1186/s12961-022-00931-1)
Supplement: Supplementary file 2 — Additional file 2. Key informant interviews—Brief. Preparatory brief document sent in advance to interview participants. [file 12961_2022_931_MOESM2_ESM.docx]

# Preparatory brief:

# Stakeholder consultation on key performance indicators & data landscape for Health System Performance measurement in Ireland

*August 12^th^ 2020*

## Aim of stakeholder consultation

Consult the views and expertise with regards to performance measurement of the Irish health system’s contributors and beneficiaries to identify key performance indicators and the current and required data landscape, by means of group interviews.

## Recall background of the project ‘Performance Accountability for the Irish Health System’

The Department of Health of Ireland requested the support of the European Commission’s Structural Reform Support Service (SRSS) for the development of an Health System Performance Assessment framework. The SRSS entails a programme of the European Commission that provides funding and expertise to support countries undergoing reforms. This request was approved and in September 2019 the project ‘Performance accountability for the Irish health system’ was launched, led by a research team of the Academic Medical Centre of the University of Amsterdam.

The project’s aims are to:

- **provide a framework** for health system performance assessment with a method for the collection, collation, and analysis of robust health outcomes data around key performance indicators in the Irish health system;
- **provide modules** within the health system performance assessment framework with measurable and quantifiable outcomes-based indicators that are linked to relevant health policies and strategies, enabling the integration of policy and reforms into a broader view of performance;
- **enhance the capacity** of the Irish authorities to produce the first HSPA report.

## Stakeholder engagement during the project phases

Throughout the project, stakeholders are engaged in all development phases to ensure solid grounding of performance measurement within the health system. The first phase encompassed stakeholder panels, to prioritize the focus of the HSPA framework. In the current phases II and III, that are conducted in parallel, stakeholders are consulted by means of group interviews to inform population of the HSPA framework with indicators and identify availability and fitness for use of data sources.

| Phase I: Developing the focus of the HSPA framework and performance reporting | Kick-off meeting  Literature review  Citizen panel  Internal stakeholder panel  **External stakeholder panel (31 January 2020)**  Policy document review |
| --- | --- |
| Phase II: Assessment of the Health Information System in Ireland | Desk research  **Consultation round through interviews** |
| Phase III: Identify domains and indicators based on best practices & priorities | Literature & policy document review  **Consultation round through interviews** |
| Phase IV: Definition of subsets of indicators for assessing specific policies | Literature & policy document review  **Consultation round through interviews** |
| Phase V: Action plan and pilot presentation of sample indicators | Synthesis of the outputs from previous phases |

##
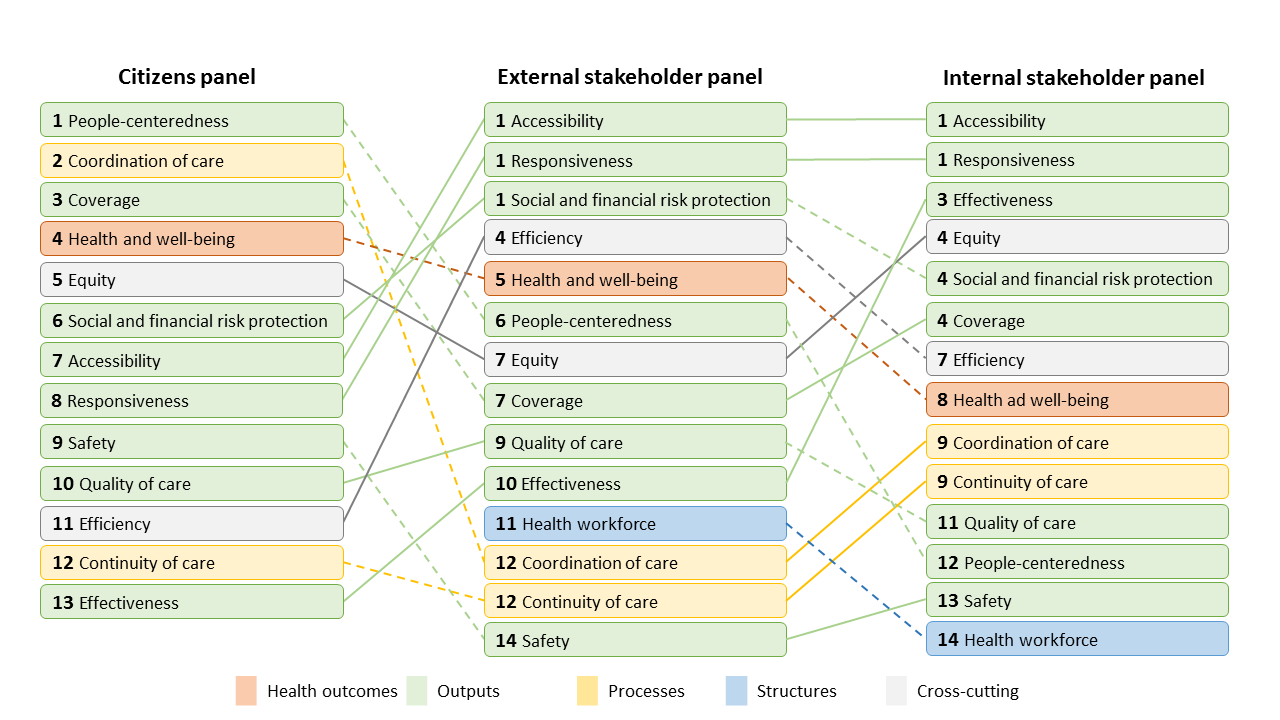
Output phase I panels

The first phase encompassed three panels, that were consulted on the priorities of health system performance in Ireland. These panels yielded similar domains, that were prioritised somewhat differently across the panels. The ‘external stakeholder’ column shows the outputs from the external stakeholder panel in which medical councils, professional associations, academia and other special interest groups participated.

## HSPA framework

Phase I resulted in a tentative HSPA Framework that was discussed with the Department of Health and an International Advisory Board of HSPA experts. The framework incorporates 16 high-level domains for performance measurement. The domains are grouped into structure, process, outputs, health outcomes and cross-cutting domains. At the bottom of the framework, the red arrows represent the non-healthcare determinants, such as housing and environmental influences, affecting the health system and its outcomes. Six modules of service delivery are included in an overlay that can be supplemented onto the performance domains.

### Purpose(s) of the HSPA Framework

The purposes of the HSPA framework are threefold:

- **To measure** performance of the delivery system (health and social services).
- **To provide information (accountability)** to the public regarding the effectiveness of policies and strategies of the DoH and HSE on overall population health;
- **To monitor** the progress of the Sláintecare reform to enable evaluation of the priority areas of the reform and ensure that the healthcare system is more responsive to the needs of the population.

### Framework


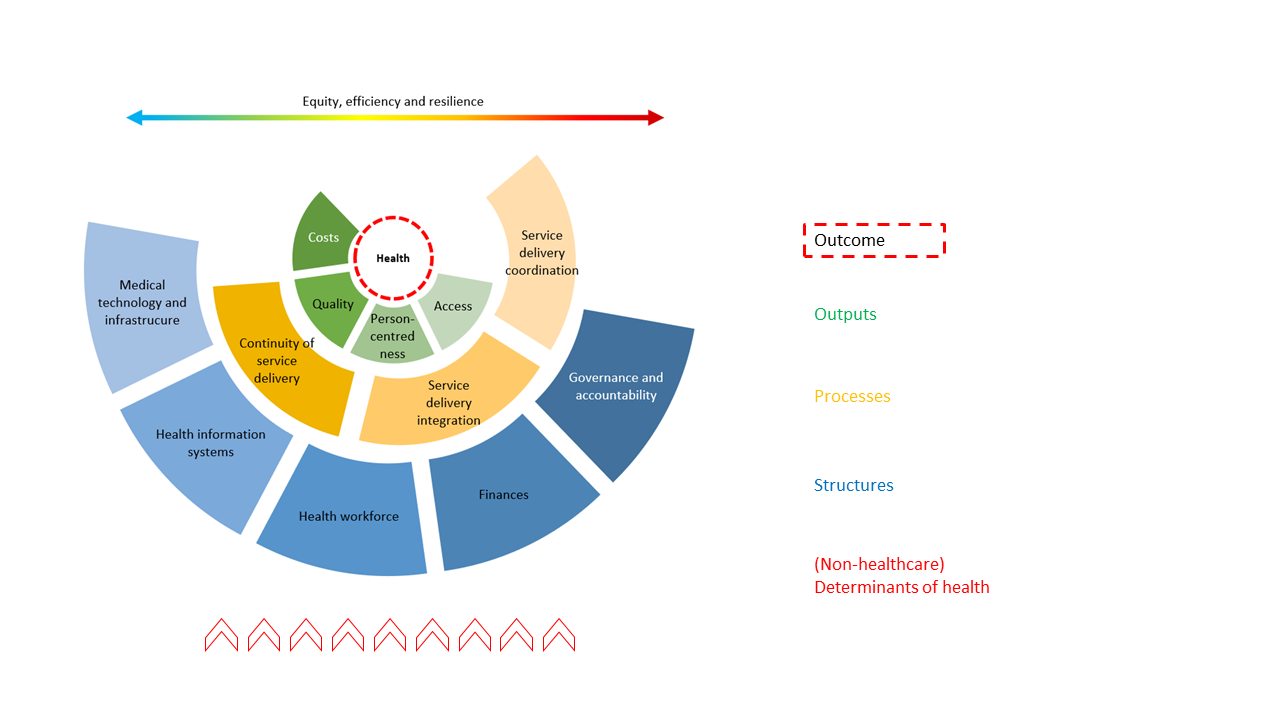


### Health services modules


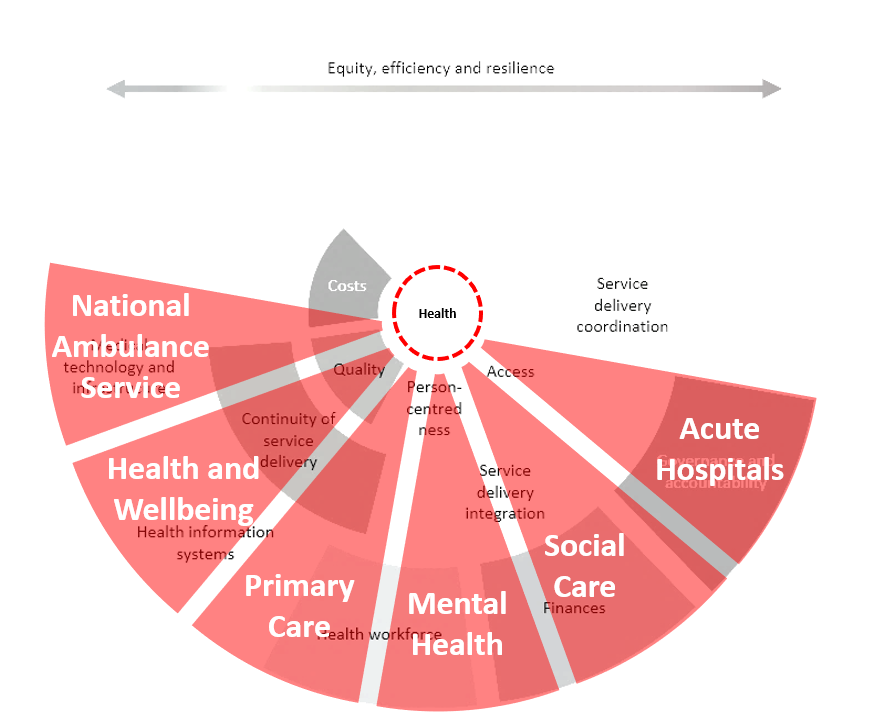


## High level performance domains

To illustrate what the high level performance domains may encompass, the domains are disaggregated to subdomains and features if applicable. This disaggregation may help to think of key performance indicators that can populate each of the domains, whether currently used or envisaged to use in the near future. Indicators are defined as a quantitative measure that provides information about a performance domain within the health system. Use is defined as the selection, sourcing, analysis and dissemination of indicators for the purpose of performance measurement. Data sources are the collections of information generated by the health system, e.g. registrations, administrative systems, surveys, that can be used as source to measure indicators.

|  | **Domain** | **Subdomains** | **Examples of features** |
| --- | --- | --- | --- |
| **Structures** | Medical Technology and Infrastructure |  | - Basic technology - Amenities |
|  | Health Information Systems |  | - Data capture - Aggregation of data - Patient platforms |
|  | Health Workforce |  | - Health workforce planning - Workforce availability - Training - Collaboration |
|  | Finances |  | - Expenditure - Payment methods - Benefit package |
|  | Governance and Accountability |  | - Priorities - Accountability arrangements - Stakeholder participation and engagement - Quality assurance mechanisms |
| **Processes** | Continuity of Service Delivery |  | - Shared care plans - Care teams - Treatment - Follow-up care - Informational continuity of care |
|  | Service Delivery Integration |  | - Comprehensive services across the care continuum - Collaboration of services |
|  | Service Delivery Coordination |  | - Care pathways - Referral system - Transition management |
|  | Overarching |  | - Strategic planning - Managing facilities - Consultation rate |
| **Outputs** | Costs |  |  |
|  | Quality | - Clinical effectiveness - Safety | - Service level quality improvement mechanisms - External accountability for quality of care - Continuous professional development |
|  | Person-centeredness | • Patient experiences  • Carer experiences  • Staff experiences | - Shared decision-making |
|  | Access | - Ability to perceive (health literacy) and approachability - Ability to seek and acceptability - Ability to reach and availability (including timeliness) - Ability to pay and affordability - Ability to engage and appropriateness | - Affordability - Timeliness |
| **Outcome** | Health & Wellbeing |  | - Burden of disease - Risk factors - Mortality |
| **Cross-cutting** | Resilience |  | - Effective and participatory leadership with strong vision and communication - Coordination of activities across government and key stakeholders - Organizational learning culture that is responsive to crises - Effective information systems and flows - Surveillance enabling timely detection of shocks and their impact - Ensuring sufficient monetary resources in the system and flexibility to reallocate and inject extra funds - Ensuring stability of health system funding through countercyclical health financing mechanisms and reserves - Purchasing flexibility and reallocation of funding to meet changing needs - Comprehensive health coverage - Appropriate level and distribution of human and physical resources - Ability to increase capacity to cope with a sudden surge in demand - Motivated and well-supported workforce - Alternative and flexible approaches to deliver care |
|  | Equity |  | - Equitable delivery of care - Equitable access |
|  | Efficiency |  | - Unnecessary procedures - Avoidable care |

## Interview approach

The following approach will be taken in conducting the group interview:

- The interview will last about one hour;
- We intend to include two to five persons in the interview;
- Two interviewers from the research team will guide the meeting;
- We will use Zoom, for which you will receive a link to join the meeting;
- With your permission, we will record the meeting for the use for this research project only.

## Guiding questions

The group interview is guided by the following questions:

- 1. What indicators are currently used for performance measurement of the Irish healthcare system within your field that you are aware of? (How) do these relate to the high level domains within the HSPA framework?

1. What data sources are available for the measurement of these indicators?
2. Is this data fit for use to measure these indicators? Why (not)?
   1. What aim is pursued with the currently measured indicators, if any?
   2. What data sources are available for current performance measurement?
   3. What initiatives, if any, are in development to measure performance in the near future?
   4. What should be aimed for by measuring performance within your field?
   5. What performance information need does your organisation have to enact on the three purposes of the HSPA framework?

*The purposes of the HSPA framework:*

- ***To measure*** *performance of the delivery system (health and social services).*
- ***To provide information (accountability)*** *to the public regarding the effectiveness of policies and strategies of the DoH and HSE on overall population health;*
- ***To monitor*** *the progress of the Sláintecare reform to enable evaluation of the priority areas of the reform and ensure that the healthcare system is more responsive to the needs of the population.*
  1. Is that information available?
  2. If so, is data fit for use for performance measurement? Why (not)?
  3. What would be needed to enable fitness for use of data? Or what – currently unavailable data – is needed?
  4. What are facilitators and barriers in the measurement of these indicators, what needs to be kept and what needs to be changed in the current data landscape to enable measurement of these indicators?
  5. Did the recent developments during the Covid-19 pandemic yield changes in performance measurement and data availability?
  6. Can you suggest a colleague, expert in your jurisdiction or network that you think should be met with in the scope of this work?
  7. Is there available work or materials of interest with regards of health system performance assessment that you are willing to share with us?
