# Supplementary material for: Status of the health information system in Ireland and its fitness to support health system performance assessment: a multimethod assessment based on stakeholder involvement
Source: Health Res Policy Syst. 2022 Nov 16;21:1. doi: 10.1186/s12961-022-00931-1 (PMC9670073; doi:10.1186/s12961-022-00931-1)
Supplement: Supplementary file 3 — Additional file 3. Stakeholder consultation workshops—Stakeholder brief and the preparatory document (excerpt). Preparatory brief document sent in advance to participants of stakeholder workshops and an excerpt of a working table used in advance and during these workshops [file 12961_2022_931_MOESM3_ESM.docx]

Stakeholder consultation workshops – Stakeholder brief and the preparatory document (excerpt)

To: Participants

Subject: **HSPA indicator workshop Tues 27 Oct 14-16:00: health outcomes cluster**

Attachment: Excel file

Dear Participants,

We are pleased to share the following details with regards to the **HSPA Indicator Workshop on Health Outcomes**. This workshop is 1 of 5 taking place in the context of the EU-funded development of a Health System Performance Assessment Framework in Ireland.

The workshop series has been organized around the 5 main clusters of the HSPA framework. An overview of the framework’s clusters and domains is enclosed. Each workshop aims to closely review a working list of indicators with representatives of its end-users: that’s you! The indicators draw from existing Irish reporting, international reporting and the scientific literature. We want to know if these indicators are useful to you, how they can be improved upon, and which ones are missed.

**Pre-workshop task**

In the attached Excel file is the current list of indicators identified pertaining to the workshop’s cluster of focus: **outcomes**. We would kindly ask you to review this list of indicators and: 1) score the indicators red/yellow/green based on the three considerations listed; 2) note comments, suggestions or modifications where needed; 3) add additional indicators you think merit discussion. We would kindly ask that you return the Excel by **end of day** **Monday Oct 26^th^ by replying to this email** in order for the responses to be summarized in advance of our workshop the next day.

**Workshop details**

**Date:** Tuesday 27 October 2020

**Time:** 14-16:00 (IRE time)

**Zoom:** link to be added

Please do not hesitate to get in touch if you have questions regarding the preparatory materials or workshop itself.

We look forward to seeing you then!

**
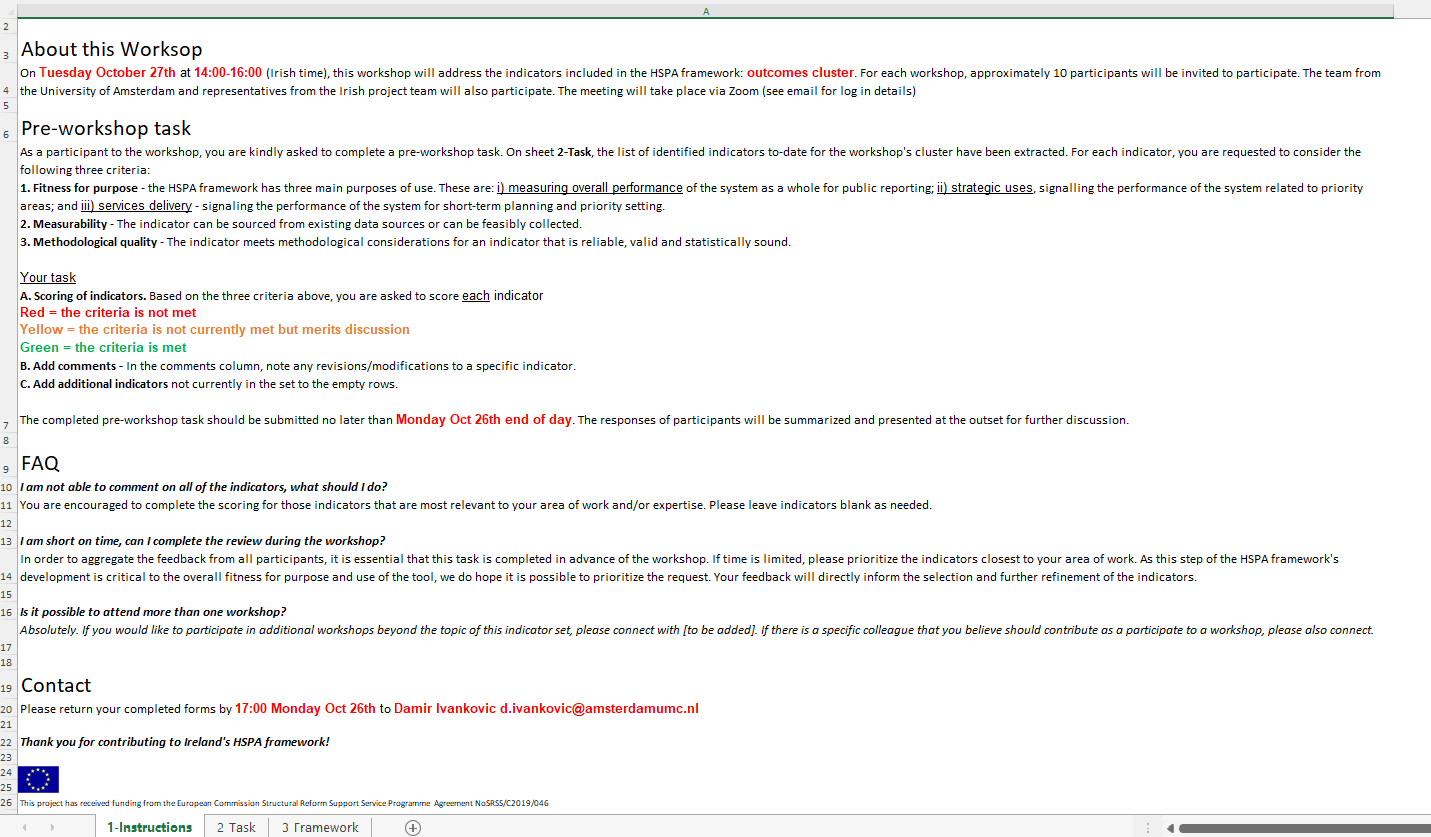

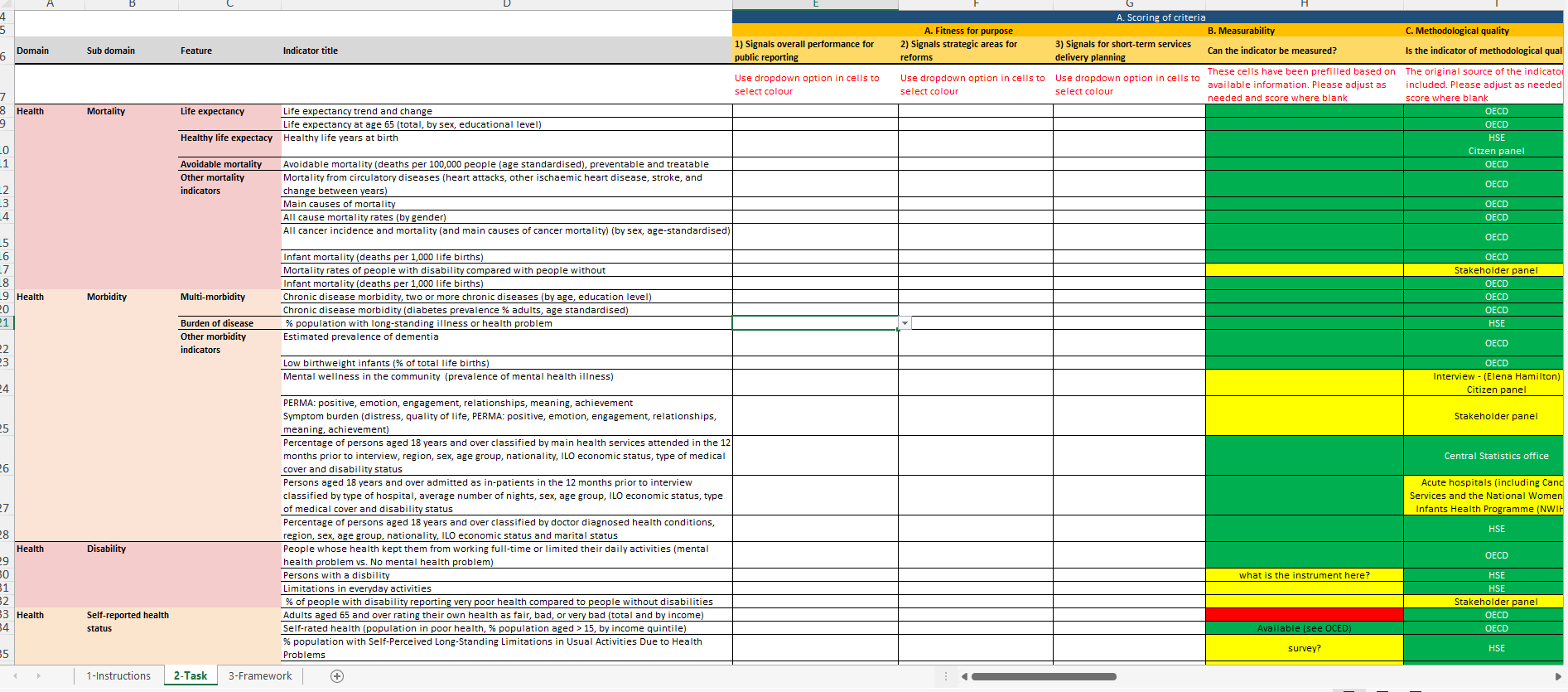
**
